# COSMO-SkyMed Image Investigation of Snow Features in Alpine Environment

**DOI:** 10.3390/s17010084

**Published:** 2017-01-04

**Authors:** Simonetta Paloscia, Simone Pettinato, Emanuele Santi, Mauro Valt

**Affiliations:** 1 Institute of Applied Physics, National Research Council, CNR-IFAC, Firenze 50019, Italy; s.pettinato@ifac.cnr.it (S.P.); e.santi@ifac.cnr.it (E.S.); 2 Avalanche Center of Arabba, Environmental Protection Agency of Veneto, CVA-ARPAV, Arabba 32020, Italy; mauro.valt@gmail.com

**Keywords:** COSMO-SkyMed, Snow Depth, backscattering, electromagnetic model, DMRT-QMS

## Abstract

In this work, X band images acquired by COSMO-SkyMed (CSK) on alpine environment have been analyzed for investigating snow characteristics and their effect on backscattering variations. Preliminary results confirmed the capability of simultaneous optical and Synthetic Aperture Radar (SAR) images (Landsat-8 and CSK) in separating snow/no-snow areas and in detecting wet snow. The sensitivity of backscattering to snow depth has not always been confirmed, depending on snow characteristics related to the season. A model based on Dense Media Radiative Transfer theory (DMRT-QMS) was applied for simulating the backscattering response on the X band from snow cover in different conditions of grain size, snow density and depth. By using DMRT-QMS and snow in-situ data collected on Cordevole basin in Italian Alps, the effect of grain size and snow density, beside snow depth and snow water equivalent, was pointed out, showing that the snow features affect the backscatter in different and sometimes opposite ways. Experimental values of backscattering were correctly simulated by using this model and selected intervals of ground parameters. The relationship between simulated and measured backscattering for the entire dataset shows slope >0.9, determination coefficient, R^2^ = 0.77, and root mean square error, RMSE = 1.1 dB, with *p*-value <0.05.

## 1. Introduction

Snow, covering up to 50,000,000 km^2^ of the Earth surface, is an important component of the global water cycle, being a vital resource of fresh water and influencing atmospheric circulation and climate at both regional and global scales. Moreover, snow distribution affects carbon cycle and greenhouse gas exchanges. 

The information required by the hydrological models for estimating the seasonal snow cover, concerns the snow spatial extent; the snowpack properties, such as grain size and albedo; the snow depth; and the snow water equivalent. These parameters significantly influence the hydrological cycle and are key elements for the monitoring of cryosphere dynamics, since snow cover changes very rapidly in time (due to snow precipitation, freeze/thaw, snow accumulation) and in space (i.e., different deposition according to the surface type). The monitoring of temporal and spatial evolution of snow cover is therefore crucial, especially in mountain areas for activities related to water resource management, the study of climate change, and weather prediction.

Satellite remote sensing allows investigating the cryosphere in remote or inaccessible areas, where conventional in-situ measurements could be difficultly available. The recent Earth observation missions as Sentinel-2 and Landsat-8, dedicated to the observation in the optical spectrum, and COSMO-SkyMed (CSK) and Sentinel-1 (S1) in the microwave bands, are very interesting for monitoring the huge temporal and spatial variability of the snow cover, due to the frequent revisit time and the high spatial resolution. However, mainly optical sensors are used so far for the operational monitoring of snow parameters from remote sensing data [1,2,3], in spite of the capability of microwave sensors to acquire data independently of day light and in adverse weather conditions. 

The potential of radars, especially Synthetic Aperture Radar (SAR), in mapping the extent of wet snow cover was investigated by using both airborne and satellite systems, demonstrating that penetration of microwaves in the snow pack depends on electromagnetic frequency and on snow conditions [4,5,6,7,8]. Models and experiments have shown that C-band data are not capable of separating snow-free areas from dry snow due to their high transmissivity, while, in some cases, the X band demonstrated sensitivity to snow depth and snow water equivalent [9]. However, wet snow can be detected by using a change detection approach, because, in this case, the backscattering is of the order 2–3 dB lower than that of dry, snow-covered soil [5,6,7,8]. The synergy between optical and SAR sensors can provide a significant improvement in the comprehension of the snow physical properties. 

Recent research pointed out that the best frequency for the monitoring of snow cover seems to be the Ku band (in the range 12–19 GHz), and an Earth Explorer for observing snow and ice was proposed to ESA, the Cold Region Hydrology High-resolution Observatory (COREH_2_O), with the simultaneous presence of two SAR systems of the X and Ku bands [10]. This mission would take advantage of the synergy between the two frequencies, since the signal at Ku band is significantly sensitive to shallow dry snow, whereas the backscatter of the X band provides higher penetration in deep snow layers and information on the underlying soil. Unfortunately, COREH_2_O has not been selected so far as an operational mission and, thus, the currently available SAR frequencies are C and X bands, only. At these frequencies, penetration is high in dry snow and very low in wet snow, thus making the estimation of the snow water equivalent a challenge [5,9,11]. Although X band alone is not yet the most suitable frequency for the retrieval of snow depth (SD) or snow water equivalent (SWE), some improvements can be expected from the analysis of consistent image datasets of the new X band SAR systems (CSK and TerraSAR-X). 

The two-fold purpose of this investigation consists of confirming the ability of integrated optical and SAR (X band) data for mapping snow cover extent, by separating snow from no-snow areas and detecting wet snow, and of evaluating the potential of CSK in providing information on snow characteristics in different physical conditions. A consistent dataset of CSK and Landsat-8 images was collected on the Cordevole basin, in the Italian Dolomites. One of the assets of this research consists of the presence of a significant amount of detailed in-situ data, which are not frequently available on snow cover areas and allowed an accurate modelization of backscatter in different snow conditions.

From the analysis of these images, the capability of X band in detecting wet snow was confirmed, as well as the synergy between CSK and Landsat-8 images for snow classification purposes. The experimental sensitivity of backscattering coefficient (σ°) to SD and SWE was then investigated during several winter seasons. Subsequently, in order to better characterize the dependence of σ° to snow parameters, a model based on the Dense Media Radiative Transfer theory was used for simulating snow cover characteristics [12,13,14]. The model (DMRT-QMS) is an implementation of the Dense Media Radiative Transfer (DMRT) theory, based on the Quasi-Crystalline Approximation (QCA) of Mie scattering of densely-packed Sticky spheres [14,15]. The model is a free code in MATLAB^®^ that was developed in the Laboratory of Applications and Computations in Electromagnetics and Optics (LACEO) at University of Washington (UW), United States and was downloaded from the site of the University of Michigan [15].

Model simulations have been carried out for interpreting the experimental results in different snow conditions characterized by various SD, snow density, and grain size. The model was applied on data collected on the selected test areas of Cherz plateau and Monti Alti di Ornella in the Cordevole basin (Veneto region, Italy), where, beside a meteorological station, detailed snow measurements were collected by the Avalanche Center in Arabba. Finally, measured σ° was correctly simulated by using appropriate intervals of ground parameters, selected in the range of the measured ones.

## 2. Materials and Methods 

### Test Areas and Satellite Images

**Figure 1:**
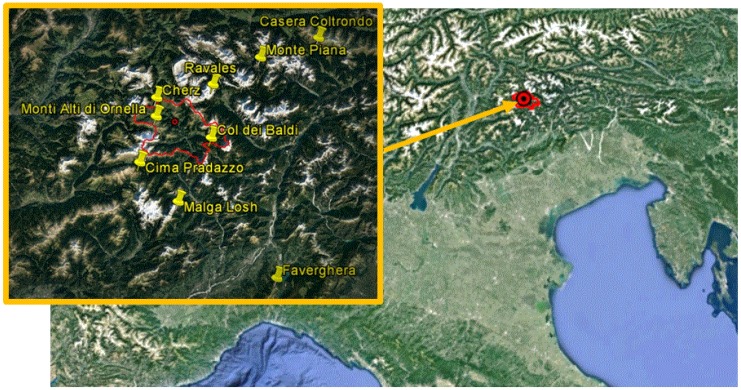
Cordevole basin in the North-eastern Italian Alps. Yellow placemarks indicate the meterological stations present on the area: (1) Col dei Baldi (1900 m a.s.l.); (2) Casera Coltrondo (1899 m a.s.l.); (3) Cima Pradazzo (2200 m a.s.l.); (4) Faverghera (1605 m a.s.l.); (5) Monti Alti di Ornella (2250 m a.s.l.); (6) Cherz (2100 m a.s.l.); (7) Ravales (2615 m a.s.l.); (8) Monte Piana (2265 m a.s.l.); (9) Malga Losh (1735 m a.s.l.). The red line represents the borders of the Cordevole basin.

The test area identified for this research (Cordevole basin) is located in Northeast of Italy on the Dolomites (Italian Alps) and it was selected because of the availability of historical and topographic data, and, in some cases, detailed in-situ measurements of snow cover [16,17] (Figure 1). Snow is usually present in the area from mid-November to mid-April; the beginning of melting/refreezing cycles usually occurs in March. Maximum snow depth in the area even reaches 250 cm. In-situ measurements are collected by automatic weather stations (AWS) displaced all over the territory of Veneto region, and the parameters acquired by each station mainly consist of snow depth and typical meteorological measurements acquired hourly (air temperature and humidity, rainfall, wind speed and direction, solar radiation). Detailed in-situ snow measurements (i.e., snow depth—SD—in cm; snow water equivalent—SWE—in mm; liquid water content, and grain shape and size, in mm) were provided by the Avalanche Center (Arabba) through conventional approaches on two homogeneous areas in the Cordevole Basin (Cherz Plateau, 2010 m a.s.l., and Monti Alti di Ornella, 2187 m a.s.l.), by digging several pits [18]. The direct measurements of snow parameters were performed periodically (weekly) and simultaneously to satellite passes, as well as every time the snow cover underwent significant changes. Snow density, *ρ*, in kg/m^3^, was indirectly retrieved from SWE and SD and represents a mean density of the entire snow layer. These data are processed in order to extract the correspondent ground data during the satellite passes. Other ancillary information, such as the digital elevation model (DEM) and land cover, is also available. DEM was derived from ASTER GDEM V2 product [19] and land cover from both the Coordination of Information on the Environment (CORINE) database [20] and NDVI computed from Landsat-8 images.

A significant satellite SAR and optical images dataset was acquired on the test area, consisting of a series of Cosmo-SKyMed [21] and Landsat-8 [22] images. Cosmo-SKyMed (CSK) acquisitions collected in different seasons by CSK1, CSK2, CSK3, and CSK4 satellite from 2012 to 2015 (for a total of 68 scenes) were delivered by the Italian Space Agency (ASI) in the framework of the COSMO-SkyMed Open Call for Science. The images were all in HH polarization and with an incidence angle (*θ*) between 30° and 40°. Landsat-8 images for 2013/2014 and 2014/2015 (for a total of four scenes) were available from the U.S. Geological Survey, from the online browser GloVis [23]. 

The database of SAR and optical acquisitions over the test area covers different seasons in order to observe no-snow soils and snow cover in various conditions. The SAR images were fully calibrated by taking into account the real size of the scattering area in each pixel according to terrain slope. The ground resolution of CSK images used for this experiment ranged from 10 to 50 m. 

In the case of SAR images acquired on mountainous zones, the physical size of the scattering area for each pixel varies according to the topography, and this effect should be taken into account in the radiometric calibration procedures. The data processing was carried out by using standard calibration procedures provided by the space agencies, implemented by commercial software (i.e., SARSCAPE^®^). The SAR data processing was carried out preliminarily on each image and it first concerned the radiometric calibration, which was performed by taking into account the local incidence angle (LIA) obtained through the DEM and the orbital parameters. These parameters were also used for generating maps of layover/shadow to be excluded from the images. Other calibration factors, such as antenna gains and specific calibration variables, were then applied to the images. The despeckling was carried out by using a multilook filter of 4 × 4 pixels, and the geocoding applying a pixel size of 10 m. Later on, all the images have been co-registered in a stack file, to allow a “pixel by pixel” comparison among the various images [24]. The cross-calibration among different images was verified by extracting the backscatter coefficients (σ°) over some natural surfaces (i.e., forests, rocks, and bare soils) used as reference targets, and successively comparing these values. Since these target areas are characterized by rather stable temporal trends of backscattering, they can be used as a reference for the cross-calibration of the images. 

The backscatter was afterward averaged over two homogeneous areas in the Cordevole Basin, namely the Cherz Plateau and the Monti Alti di Ornella sites having a surface area of 150 × 150 m^2^ and 180 × 150 m^2^, respectively, and where detailed in-situ snow measurements were available. 

## 3. Results

### 3.1. Preliminary Image Classification

A preliminary multi-temporal analysis was performed on CSK and Landsat-8 images, when simultaneous acquisitions are available, with the aim of confirming the results obtained at C band in previous works for the identification of different surface classes (i.e., snow/no-snow/wet-snow). Snow is usually defined as “‘wet’ in case that it holds free liquid water, and ‘dry’ when it doesn’t” as it has been specified in [25].

The classification of the surface cover as “snow”/”no snow” was obtained by using Landsat-8 image and the NDSI approach [1,2,3], and subsequently, the estimate of wet snow was performed by applying the well-known Nagler threshold algorithm [6]. The latter classifies snow as wet, due to the presence of liquid water, when the backscattering coefficient, σ°, is 3 dB lower than a reference value collected in no-snow conditions. This procedure was mainly applied so far to ERS1-2, RADARSAT, and ENVISAT/ASAR data (i.e., C-band sensors) [6,26,27]. 

With the launch of COSMO-SkyMed (CSK) and TerraSAR-X (TSX) in 2007, data from spaceborne X band sensors are largely available, and some studies regarding snow monitoring by using these data begun to be published. Venkataraman et al. [28] applied the ratio method, using a threshold of 3 dB, to TSX images to obtain wet snow maps in the Gangotri glacier region (Himalaya), as well as Schellenberger et al. [29], which used CSK images on a test site in South Tyrol (Italian Alps).

**Figure 2:**
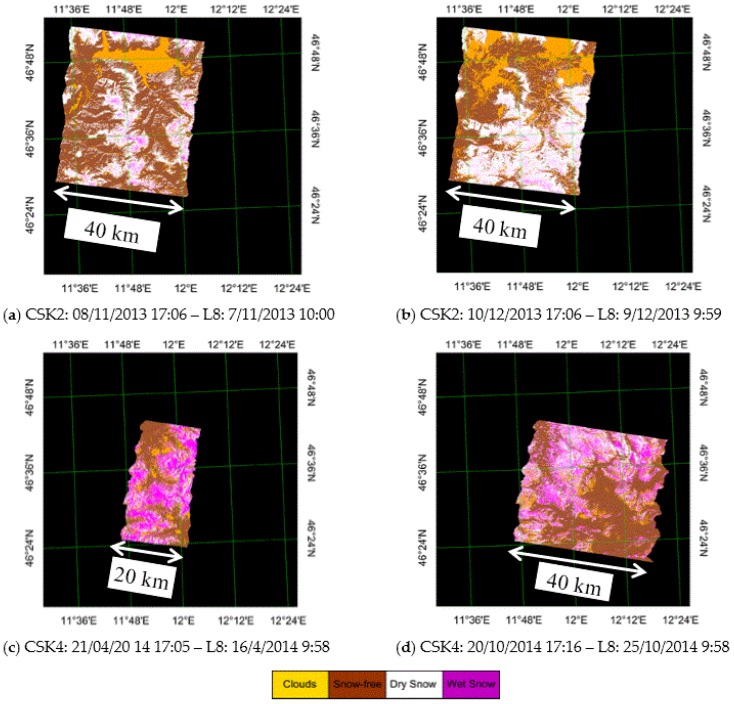
Four overlapped couples of CSK and L8 images of the Cordevole area acquired in different seasons, identifying the snow cover area and separating dry from wet snow. (**a**) November 2013; (**b**) December 2013; (**c**) April 2014; (**d**) October 2014. The size of the images is roughly 40 km × 40–50 km, except in (**c**) where the dimensions are smaller, 20 km × 40 km. Legend: white = dry snow, magenta = wet snow, brown = snow-free soil, yellow = clouds.

We, in our turn, have applied this threshold algorithm to CSK images [6,8] collected on Cordevole area. Both the optical and SAR images have been acquired by the same period, very close in time, as it can be observed in Table 1, where also the reference summer images are listed. No snowfall occurred between the two observations. The maps represented in Figure 2 show that in November and December 2013 most snow cover was dry (white zones), whereas in April and October 2014, most snow cover was wet (magenta zones). These results are in line with the season and the temperature measured at the meteorological stations present on the area, confirming that X band σ° also is able to provide the identification of wet snow, as it was already pointed out in the C band [6,8,17]. 

### 3.2. Sensitivity of Backscatter to Snow Characteristics

**Figure 3:**
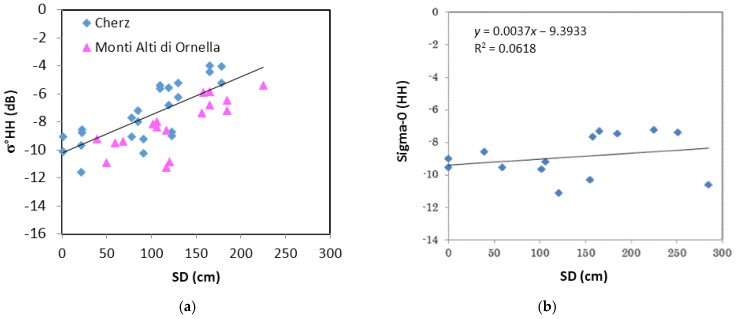
(**a**) σ° at X band in HH polarization vs. SD collected in Cordevole basin (Cherz and Monti Alti di Ornella) during winter seasons, 2012–2013 and 2014–2015. The obtained regression line is σ° = 0.027SD – 10.24 with R^2^ = 0.65. (**b**) σ° at X band in HH polarization vs. SD collected in Cordevole area during winter 2013–2014.

After this analysis, which confirmed the sensitivity of σ° at X band to wet snow areas, the behavior of backscatter at X band (CSK) as a function of snow depth (SD, in cm) was investigated. In the diagram of Figure 3a, σ° in HH polarization is directly related to SD measured at several snow stations on the Cordevole basin (Cherz and Monti Alti di Ornella) in winter seasons between 2011 and 2015. Only data collected in dry snow conditions have been considered, according to the air temperature (T_air_ < 0°) measured at the meteorological stations. The increasing trend of σ° is clear, especially at SD ≥ 50 cm, with a determination coefficient (R^2^) of about 0.65. The slight difference between the two datasets in terms of average backscattering values can be attributed to variations in the local incidence angle. Similar trends have also been confirmed by observations carried out at VV polarization in previous winter seasons [9], and in another Italian snow area (Bardonecchia) in the Northwest Italian Alps in 2011–2012 [30]. 

However, the sensitivity of X band backscatter to SD has not always been observed, since it depends not only on SD but also on other parameters, such as grain size, snow density, and temperature. An example of this controversial behavior is shown in Figure 3b, where σ° at X band in HH polarization, still acquired on Cordevole area in winter 2013–2014, is represented vs. SD. We can note that, in spite of the presence of consistent snow depth values derived from the local snow stations, the variations of σ° on the area is almost negligible.

A clear interpretation of the behavior of σ° is in this case problematic, considering the difficulties in obtaining in-situ detailed snow parameters for each date, and the extrapolation of σ° trend on a large scale cannot be so trivial. At a first glance, one of the reasons for this different behavior of σ° vs. SD in subsequent winter seasons could rely on the snow temperature. Winter 2013–2014 was indeed a rather mild winter, characterized by heavy snowfalls and relatively high air temperatures, i.e., close to 0°, measured at the local meteorological stations. This fact could have produced a slight melting of the very first snow layer, causing, therefore, a σ° behavior similar to wet snow, although the in-situ measurements of air temperature did not point out this phenomenon. Such high temperatures could have nevertheless also affected other characteristics of snow cover, such as grain size, snow density, and so forth. In the case of a warm winter, in fact, the snow grains increase in size due to the melting and refreezing cycles, and ice crusts could be formed within the snow cover (with snow density even higher than 500 kg/m^3^), both factors definitely affecting the backscattering [31]. 

Therefore, in order to investigate more in-depth the behavior of backscattering related to snow characteristics, and to confirm these preliminary considerations, a model based on Dense Medium Radiative Transfer theory (DMRT) was used for simulating backscattering and the results are shown in the following sections. 

### 3.3. DMRT Model Simulations with Experimental Data

The model is an implementation of the dense media radiative transfer (DMRT) theory, applying the scattering model of QCA (Quasi-Cristalline Approximation) Mie of densely packed Sticky spheres (DMRT-QMS). The DMRT describes the scattering in a medium with particle fractional volume >10% (independent scattering is not valid). DMRT equations are derived from Dyson’s equation under the QCA approximation and from the Bethe Salpeter equations under the ladder approximation of correlated scatterers. The correlation of particle position was described by the pair distribution function of the Percus–Yevick approximation. To solve the DMRT equations, the diffuse intensities have been decomposed into Fourier series in the azimuthal direction. Each harmonic is solved by the eigenquadrature approach. No particle size distribution is used in this work [12]. The model is a free code in MATLAB^®^ that has been developed in the Laboratory of Applications and Computations in Electromagnetics and Optics (LACEO) at University of Washington (UW), Seattle, WA, USA [15]. The model was used for investigating the dependence on σ° to snow parameters in different conditions of snow cover and it was run for a single snow layer [12,13,14]. 

The physical input parameters of the model are grain radius (*r*, in mm), snow density (*ρ*, in kg/m^3^), and stickiness of particles (0.1), soil permittivity, and surface roughness parameters (height standard deviation, in cm), some of which derived from in-situ measurements. The soil contribution was accounted for by using the Oh model [32]. The dielectric constant of soil has been assumed equal to the one of frozen soil (dielectric constant ≈ 6 + j2) depending on the air temperature measured at the meteorological stations. 

**Figure 4:**
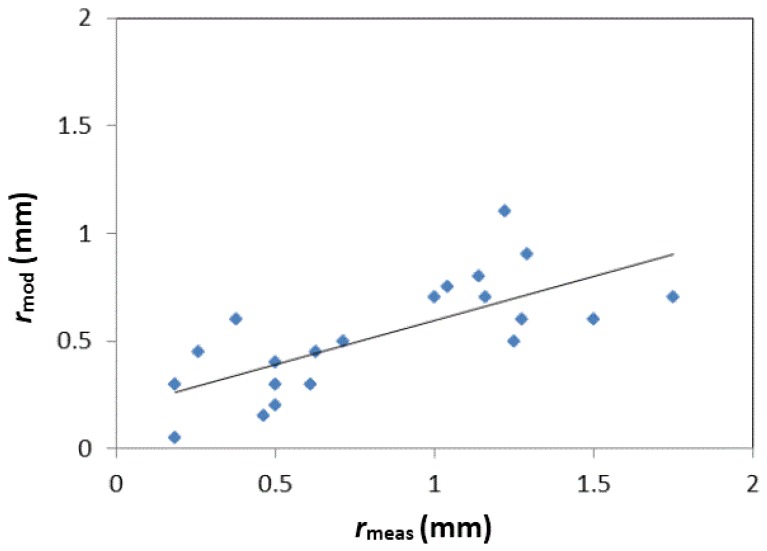
Simulated grain radius (*r*_mod_, in mm) as a function of the measured one (*r*_meas_, in mm).

Two key problems of the DMRT model are the estimate of the actual values of grain radius and stickiness, which contribute to the total scattering from snow. The stickiness was found to have a smaller influence on the model than the grain radius and was therefore assumed to be constant and equal to 0.1. Variations of stickiness between 0.1 and 0.3 provide in fact negligible variations in backscatter. Since the relationship between measured grain size and equivalent radius, required as model input, is challenging, the grain radius, along with the snow temperature (T_snow_), was kept free in the interval of experimental data (i.e., grain radius 0.05–1.5 mm, T_snow_ 230–273 K). The values of grain radius, which allowed the minimization of the error between measured and simulated backscatter (*r*_mod_), were compared with the in-situ measurements (*r*_meas_), in order to check if a clear scaling relationship could be retrieved. The diagram of Figure 4 was obtained, with the following relationship: *r*_mod_ = 0.41*r*_meas_ + 0.18 (R^2^ = 0.53). Although the correlation between the two parameters does not show a very high determination coefficient, the trend is clear and makes the assumption of using the grain radius as a free parameter in the model reliable, taking into account an almost constant scaling between the two radiuses.

The list of parameters and their range, used as inputs to model simulations, are shown in Table 2. 

A sensitivity analysis was carried out at X band (9.6 GHz) by using the DMRT-QCA model, in order to check the importance of snow parameters on the main model parameters, i.e., scattering, extinction, and absorption coefficients. The extinction coefficient, *κe,* which represents the total loss of dry snow, is defined as the sum of the absorption and scattering loss: *κe* = *κa* + *κs,* where *κa* is the absorption and *κs* the scattering coefficient. The absorption coefficient *κa* is defined as: *κa* = 2*k*_0_Im[(*ε_g_/ε*_0)_^1/2^], where *k*_0_ is the wave number of free space; *ε_g_* is the quasi-static value of the dielectric constant of dry snow, and *κs* is deduced from the phase matrix components [33]. At high microwave frequencies, scattering generally dominates over absorption, according to Mie theory [34]. 

**Figure 5:**
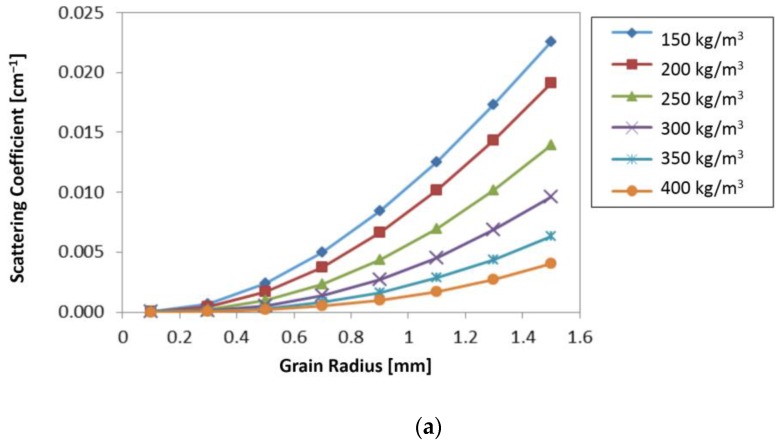

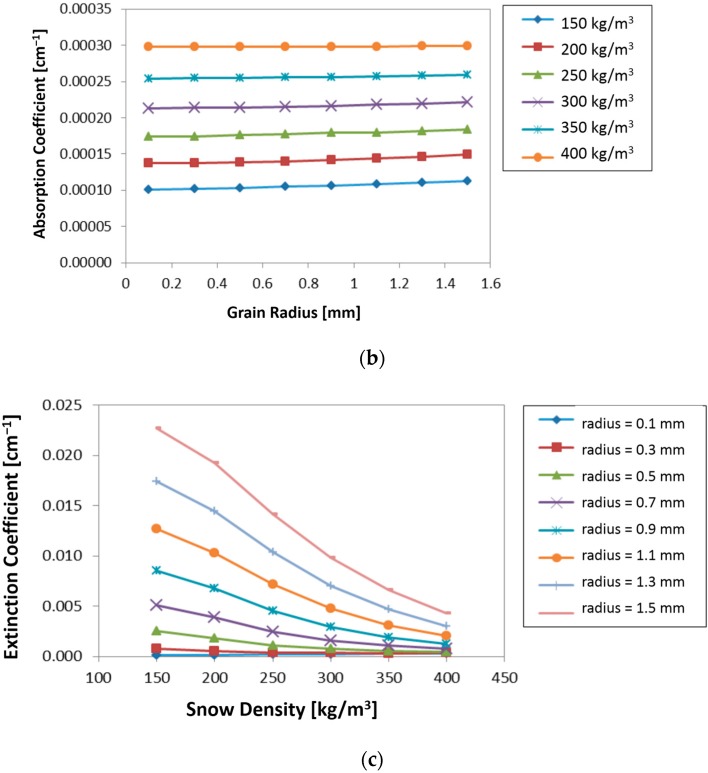
(**a**,**b**): Scattering and absorption coefficients, respectively, as a function of grain radius (in mm) for different values of snow density (150–400 kg/m^3^); (**c**) extinction coefficient vs. snow density for different values of grain radius (0.1–1.5 mm).

In Figure 5, the scattering and the absorption coefficients were shown as a function of grain radius (in mm) for different values of snow density (*ρ* = 150–400 kg/m^3^). In the same figures, the extinction coefficient was shown vs. snow density for different values of grain radius (0.05–1.5 mm). The scattering coefficient tends to increase as the grain radius increases, more for low than for high snow densities. It can be noted that at *ρ* > 300 kg/m^3^ *κs* becomes almost insensitive to grain radius, whereas the absorption coefficient seems to be insensitive to grain size and increases as the snow density increases. The extinction coefficient represented in Figure 5 vs. *ρ*, shows a decreasing trend only for high values of grain radius (>0.7 mm), while at low values of grain radius is almost constant. This sensitivity analysis confirms that the behavior of σ° can change dramatically as grain radius and snow density—which affect the absorption, scattering, and extinction coefficients in opposite ways—vary.

After this theoretical sensitivity analysis, the experimental snow measurements (SD, SWE, and *ρ*), collected on Cherz plateau and Monti Alti di Ornella, were used as inputs to the model to simulate the actual σ° (at HH polarization and incidence angle *θ* = 33°) vs. both ρ and grain radius. Since the average local incidence angle of the site was about 33°, this value was used into the DMRT. The snow temperature (T_snow_, 230–273 K) and grain radius (*r*, 0.05–1.5 mm) are kept free in the interval of experimental data.

**Figure 6:**
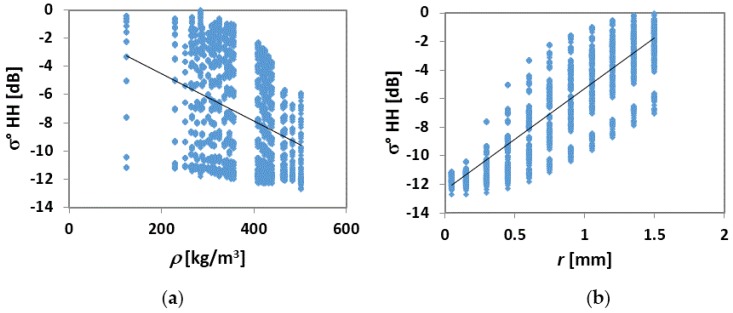
(**a**) σ° (HH) values vs. ρ derived from different SD values of the dataset collected on Monti Alti di Ornella. The obtained regression line is σ°= −0.017*ρ* − 1.16, R^2^ = 0.14; (**b**) σ° vs. grain radius, *r*. The regression line is σ° = 7.1*r* − 12.4, R^2^ = 0.78.

**Figure 7:**
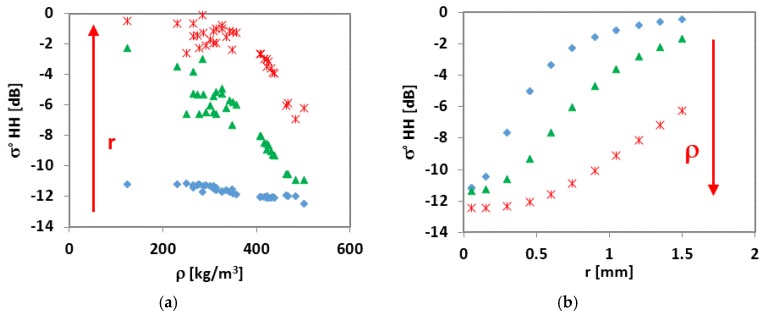
(**a**) σ° values vs. ρ for selected values of r (diamonds: 0.05 mm; triangles: 0.075 mm; asterisks: 0.15 mm); (**b**) σ° vs. grain radius (*r*) for three values of ρ (diamonds: 124 kg/m^3^; triangles: 300 kg/m^3^; asterisks: 500 kg/m^3^).

In Figure 6, σ° values were generated vs. *ρ* derived from different SD values of the dataset collected on Monti Alti di Ornella (a) and grain radius (*r*) (b). We can note that, although with a great spread of data, σ° tends to decrease with increasing *ρ*, and increase with increasing *r*, although the latter parameter seems to influence the backscatter in a more consistent way with respect to *ρ*, as it is pointed out by the higher slope and determination coefficient (R^2^ > 0.75) of the regression line (Figure 6b). By using selected values of both grain radius (i.e., 0.05 mm, 0.75 mm, and 1.5 mm) and *ρ* (i.e., 124 kg/m^3^, 300 kg/m^3^, and 500 kg/m^3^), respectively, the diagrams are transformed into those of Figure 7a,b. The trends of σ° definitely decrease as a function of *ρ*, and increasing as a function of *r* and are well separated for each selected value, thus confirming the theoretical trends of Figure 5. At smaller grain size (0.05 mm) and higher *ρ* (500 kg/m^3^), the trend of σ° is instead rather flat (Figure 7a,b).

**Figure 8:**
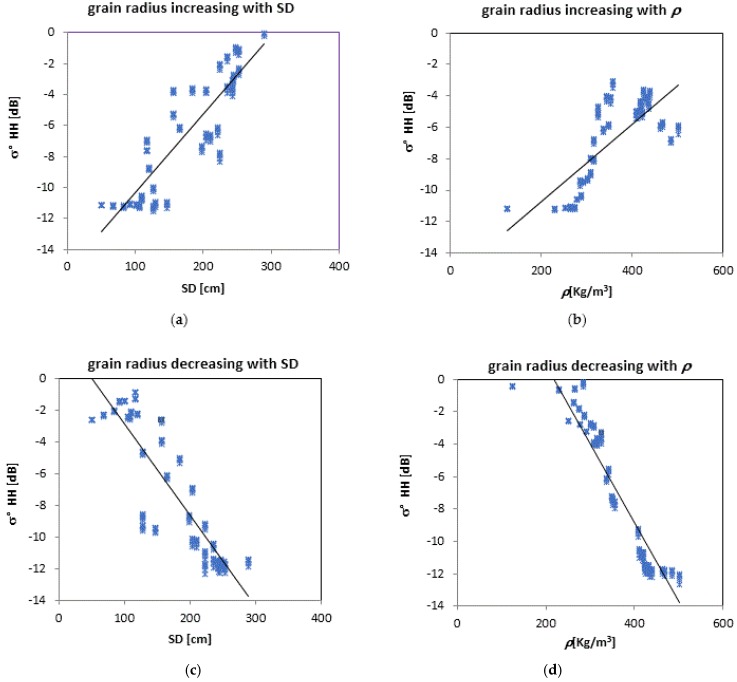
σ° (HH pol.) as a function of SD and ρ obtained by assuming a grain radius, *r*, increasing or decreasing with SD (**a**,**c**) and *ρ* (**b**,**d**), respectively.

For better assessing the effect of grain radius on model simulations, a more in-depth analysis was carried out by generating values of σ° as a function of SD and ρ by increasing or decreasing the grain radius (Figure 8). Simulations have been carried out for a subset of data with respect to the dataset of Figure 3, since snow in-situ measurements were available in some dates only. In the first two diagrams (and b) the simulations were performed assuming *r* linearly increasing with SD (left) and ρ (right), within the selected intervals of experimental data, by using the following relationships: *r* = 0.0045*ρ* − 0.7817 (a), and *r* = −0.0045*ρ* + 2.3642 (b). In the last two diagrams, *r* was assumed to be linearly decreasing as both SD (c) and ρ (d) increase, according to the following relationships: *r* = 0.0069SD − 0.3911 (c) and *r* = −0.0069SD − 1.9863 (d).

From the analysis of these diagrams, we can see that σ° definitely increases with both SD and *ρ*, when the grain radius increases. On the other hand, we observe a systematically opposite trend of σ° if the grain radius decreases as SD and *ρ* increase. These behaviors could explain the different trends of σ° vs. SD shown in Figure 3. The insensitivity of σ° as SD increases can be attributed indeed to a corresponding decrease of average r and/or an increase of *ρ*. 

Hence, the variations of grain size significantly affect the backscatter simulations, as it can be noted from the obtained regression lines, which show rather high determination coefficients:

- By increasing grain radius: σ° = −0.058SD + 2.89 (R^2^ = 0.78); σ° = −0.025ρ − 15.64 (R² = 0.58);
- By decreasing grain radius: σ° = 0.051SD − 15.43 (R^2^ = 0.77); σ° = −0.049ρ + 10.64 (R^2^ = 0.89).

**Figure 9:**
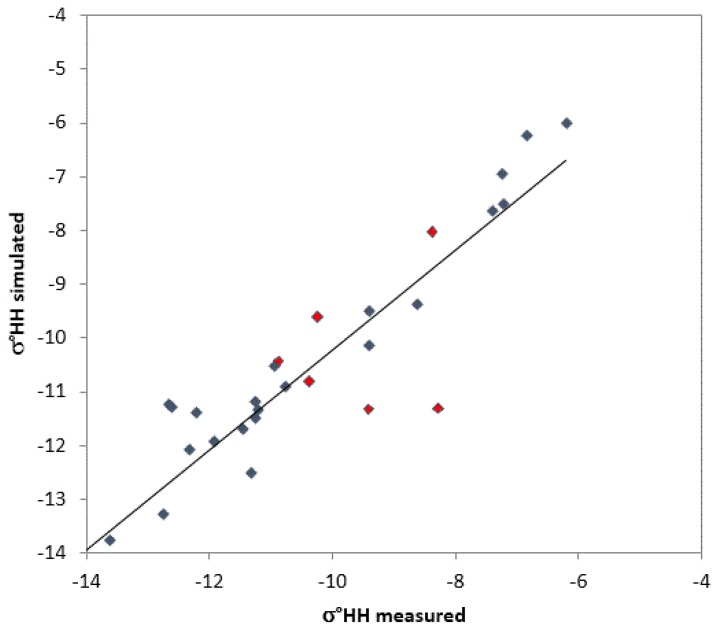
Simulated σ° (HH pol.) as a function of measured σ°. The obtained regression equation is σ°_sim_ = 0.93σ°_meas_ − 0.96 (R^2^ = 0.77, RMSE = 1.1 dB, *p*-value < 0.05). The backscattering values not sensitive to SD are shown in red (see Figure 3b).

Finally, a direct comparison between simulated and measured σ° is shown in Figure 9, where it can be observed that most of the σ° values are correctly simulated in various snow cover conditions, although the model only considers dry snow over frozen soil, and it is not able to take into account ice crusts inside the snow cover. σ° values, which did not show any correlation to SD (i.e., Figure 3b), are marked in red. 

The statistical parameters of the regression for the entire dataset are slope >0.9, R^2^ = 0.77, RMSE = 1.1 dB, with *p*-value <0.05. This general relationship has been obtained considering both cases in which σ° values increasing with SD and σ° values are insensitive to SD. These data refer to a subset for which in-situ snow measurements were available and therefore do not overlap completely with the dataset shown in Figure 3. The minimization between simulated and experimental σ° values was carried out on the basis of the best value of grain size, which has been considered as a free parameter. 

## 4. Discussion and Conclusions

Simultaneous image acquisitions from optical and SAR satellites (Landsat-8 and Cosmo-SkyMed) on Italian Alps (Cordevole basin) confirmed the capability of these sensors in classifying snow cover, by identifying snow-free and snow cover areas and in detecting wet snow. These preliminary results showed the potentiality of synergy between different satellite sensors in detecting snow conditions and the possibility of equally using X or C band SAR. 

The sensitivity of X band σ° to the dry snow depth is instead controversial, showing different behaviors depending on the snow characteristics related to the winter season. 

Thus, theoretical model simulations, based on DMRT-QMS model, have been carried out for interpreting the experimental results. Ancillary information (i.e., digital elevation model, meteorological and in-situ snow data, the latter collected by the Avalanche center in Arabba, Italy) have been used to correctly implement the theoretical model. By using DMRT-QMS and snow in-situ data, the effect of grain size and snow density—which affect the backscatter in an opposite way, beside SD and SWE—were investigated. As expected, grain size significantly affects the backscattering variations. Although the grain radius was considered a free parameter in the interval of experimental data (i.e., 0.05–1.5 mm), we have found a good correlation between measured and simulated grain radius, with a constant scaling. 

Simulations showed that measured σ° values have been correctly simulated, with R^2^ > 0.7 and RMSE = 1.1 dB, in both cases of backscattering decreasing and increasing with snow depth, by using selected intervals of ground parameters, in the range of the measured ones. Although the model is not able to take into account ice crusts inside the snow cover, and it only considers dry snow over frozen soil, backscattering was correctly simulated in most cases.

From the results presented in the paper, one of the crucial points observed is that X band σ° is significantly influenced by grain radius and density in an opposite way. The behaviors of these two parameters could explain the different trends of σ° vs. SD shown in Figure 3. The insensitivity of σ° as SD increases can be attributed indeed to a corresponding decrease of average grain radius or/and an increase of density. Metamorphism processes occurring in the snow cover due to sudden variations of air temperature and heavy snowfalls can lead to these concurrent variations of snow parameters. 

This brings us to the conclusion that, in order to minimize the uncertainties on the correct σ° trend retrieval, at least one these two unknowns (i.e., snow density and grain size) should be measured or derived from other observations. Actually, an attempt to retrieve snow grain size from optical satellite data was performed in [35], while the retrieval of snow density maps seems harder to perform. This confirms that the synergy between optical and SAR sensors should be enhanced, taking advantage from the different observation capabilities of each sensor. 

Further investigations will be carried out on the basis of SAR images and available snow data to interpret more in-depth the different sensitivities of backscatter to snow depth and to better quantify how snow depth, density, and grain size affect the σ°. The final goal should be to exploit the synergy of optical and SAR data (at X band) to investigate snow characteristics and retrieve snow parameters, by providing indications for operational use of CSK and optical sensors on snow. Thus, since each sensor has its own capabilities in snow monitoring, these aptitudes could be used in a synergic way, by using optical sensors for identifying snow cover area and providing indications on the grain size, although in cloud free conditions only, SAR sensors (at both C and X band) for discriminating between wet and dry snow/soil, and, finally, X band backscatter for retrieving snow parameters in dry snow conditions.

## Figures and Tables

**Table 1:** Couples of CSK and L8 images acquired in the Cordevole area. Also reference summer CSK images are listed. Dates and hours of acquisition are shown.

CSK Images	Reference CSK Images	L8 Images
CSK2 08/11/2013 – 17:06	CSK2 24/09/2012	07/11/2013 – 10:00
CSK2 10/12/2013 – 17:06	CSK2 24/09/2012	09/12/2013 – 9:59
CSK4 21/04/2014 – 17:05	CSK4 19/07/2012	16/04/2014 – 9:58
CSK4 20/10/2014 – 17:16	CSK4 19/07/2012	25/10/2014 – 9:58

**Table 2:** List of the DMRT-QCA model inputs.

Snow Parameters	Min	Max
Snow Density (*ρ*, kg/m^3^)	200	350
Snow Depth (SD, cm)	40	200
Grain Radius (r, mm)	0.05	1.5
T_snow_ (K)	230	273
Incidence angle (*θ*, °)	≅33	
**Soil Parameters**		
Soil permittivity	6 + j2	
Height standard deviation (cm)	0.5	
